# Real-time myoelectric control of a multi-fingered hand prosthesis using principal components analysis

**DOI:** 10.1186/1743-0003-9-40

**Published:** 2012-06-15

**Authors:** Giulia C Matrone, Christian Cipriani, Maria Chiara Carrozza, Giovanni Magenes

**Affiliations:** 1 Dipartimento di Ingegneria Industriale e dell’Informazione, Università degli Studi di Pavia, Via Ferrata 1, 27100, Pavia, Italy; 2 The BioRobotics Institute, Scuola Superiore Sant’Anna, V.le Piaggio 34, 56025, Pontedera, Italy

## Abstract

**Background:**

In spite of the advances made in the design of dexterous anthropomorphic hand prostheses, these sophisticated devices still lack adequate control interfaces which could allow amputees to operate them in an intuitive and close-to-natural way. In this study, an anthropomorphic five-fingered robotic hand, actuated by six motors, was used as a prosthetic hand emulator to assess the feasibility of a control approach based on Principal Components Analysis (PCA), specifically conceived to address this problem. Since it was demonstrated elsewhere that the first two principal components (PCs) can describe the whole hand configuration space sufficiently well, the controller here employed reverted the PCA algorithm and allowed to drive a multi-DoF hand by combining a two-differential channels EMG input with these two PCs. Hence, the novelty of this approach stood in the PCA application for solving the challenging problem of best mapping the EMG inputs into the degrees of freedom (DoFs) of the prosthesis.

**Methods:**

A clinically viable two DoFs myoelectric controller, exploiting two differential channels, was developed and twelve able-bodied participants, divided in two groups, volunteered to control the hand in simple grasp trials, using forearm myoelectric signals. Task completion rates and times were measured. The first objective (assessed through one group of subjects) was to understand the effectiveness of the approach; i.e., whether it is possible to drive the hand in real-time, with reasonable performance, in different grasps, also taking advantage of the direct visual feedback of the moving hand. The second objective (assessed through a different group) was to investigate the intuitiveness, and therefore to assess statistical differences in the performance throughout three consecutive days.

**Results:**

Subjects performed several grasp, transport and release trials with differently shaped objects, by operating the hand with the myoelectric PCA-based controller. Experimental trials showed that the simultaneous use of the two differential channels paradigm was successful.

**Conclusions:**

This work demonstrates that the proposed two-DoFs myoelectric controller based on PCA allows to drive in real-time a prosthetic hand emulator into different prehensile patterns with excellent performance. These results open up promising possibilities for the development of intuitive, effective myoelectric hand controllers.

## Background

A successful replacement of the natural hand with an artificial prosthesis may be achieved through the use of a dexterous anthropomorphic hand, controlled and perceived in a close-to-natural way by means of an intuitive human–machine interface. One of the conditions required for a satisfactory and usable prosthesis is that the interface effectively decodes the efferent motor commands dispatched by the amputee’s brain to perform the desired actions with accuracy and acceptable cognitive effort.

Traditional myoelectric prostheses (e.g. Otto Bock SensorHand, Motion Control hand) have just one or two degrees of freedom (DoFs), sequentially operated through electromyographic (EMG) signals picked-up by surface electrodes from the residual limb muscles. Although these devices are extremely robust and require low burden for the user in learning their operation, they offer poor cosmetic appearance and limited functionalities. Similarly, the recently marketed multi-fingered prototypes (like the Touch Bionics i-Limb and RSL Steeper BeBionic) are still based on a traditional two-input EMG controller used to open and close all the fingers of the hand together. This drawback is due to the difficulties of reliably using—in the practice—more than two EMG channels simultaneously [1]. Indeed, despite the recent achievements reached by researchers in developing multi-DoF prostheses (e.g. the SmartHand [2], the VU hand [3], the DARPA RP 2009 *Intrinsic Hand*[4]), these are not yet used in the clinical practice due to the lack of adequate interfaces with the user.

The most successful and common method employed for prostheses control is based on EMG processing [5]; in order to myo-electrically control a dexterous prosthesis it is necessary to map EMG signals (corresponding to different muscle contractions) to the different existing DoFs, using a suitable control algorithm [6]. This is a multiple input, multiple output (MIMO) problem where the goal is to best map the EMG inputs to the outputs in the prosthesis (i.e. the number of actuators). EMG control techniques can be divided in two categories: *pattern recognition* and *non-pattern recognition* based [7]. In research, sophisticated algorithms (usually running off-line) implement pattern recognition: features extracted from the EMG signals (e.g. mean absolute value (MAV), root mean square (RMS), zero crossing, or frequency domain features; for a review of the features see [7] and [8]) are used to decode different muscular contractions, using various classification algorithms (e.g. multilayer perceptrons, fuzzy techniques, wavelets, linear discriminant analysis) [9-16]. Non-pattern recognition control, traditionally used in the clinical practice [17], includes proportional control, threshold control, onset analysis and finite state machines. The number of functions that can be controlled by non-pattern recognition techniques is limited in comparison to pattern recognition based ones but, in general, non-pattern recognition controllers offer greater reliability. They have a simpler structure and have been mostly deployed in ON/OFF or proportional control. In particular, in proportional control the strength of muscle contractions controls the prosthesis speed or force [7].

In order to achieve tangible improvements in this field, the fundamental issues to be tackled are: (1) how to increase the voluntary controlled dexterity (i.e. the number of controllable DoFs) and, at the same time, (2) how to provide the amputee with an intuitive and effective way for controlling his/her artificial limb. With the aim to address such problems, this study presents a control method suitable for multi-fingered prostheses, based on surface EMG and bio-inspired to *muscle synergies* involved in natural motor coordination.

A functional muscle synergy represents the elementary unit of motor behaviour and is defined as a pattern of co-activation of muscles recruited by a single neural command signal [18]. Back in the 1960s, Nikolai Bernstein proposed the existence of muscle synergies as a neural strategy for simplifying the control of multiple DoFs [19]. More recently, Santello *et al.*[20] demonstrated that this same strategy is effective also in the case of hand control and that, during grasps, some DoFs of the hand are tightly correlated. In particular, in that experiment, subjects were asked to shape the hand as if to grasp and use a large number of familiar objects, while static finger postures were measured by means of a data-glove; Principal Components Analysis (PCA) showed that the first two components could account for 80% of the variance, implying a substantial reduction from the 15 DoFs that were recorded. Hence, they demonstrated that only few synergies are involved in the control of hand posture (even though this mechanism is coupled with a finer control mechanism providing for small, subtle adjustments) [20]. Drawing inspiration from this biomechanical/neuromuscular behaviour, in our previous work we developed a control algorithm based on PCA able to map two continuous control inputs into continuous multiple outputs (motors) of a robotic hand [21]. In addition we demonstrated the ability of the system to achieve stable grasps when the two control inputs were virtually generated. If extended to upper-limb prosthetics, such an approach could allow amputees to control multi-DoF hands using an extremely reduced number of myoelectric channels. Remarkably, the approach holds the potential to overcome the MIMO problem (i.e. how to map a reduced number of inputs into a large number of outputs), as it could allow successful control of hands—if mechanically capable—into 80% of the natural hand postures by just modulating two input channels.

Previous works, aimed to control dexterous hands or robotic grippers, exploited the concept of synergies in order to reduce the dimension of the problem. Particularly, the PCA technique, usually employed in prostheses control algorithms to preprocess (decorrelate) EMG signals [22] or to reduce features dimensionality [10,23], has also been exploited in robotics to implement automatic grasp planners [24,25] or even to design the mechanical architecture of an anthropomorphic hand [26].

This paper presents the first work on the real-time prehension control of a robotic hand employing a two differential channels (four electrodes) EMG acquisition system and a PCA-based controller, operated by 12 able-bodied subjects. Participants volunteered in experimental tasks consisting in (i) *grasping*, (ii) *transporting* and (iii) *releasing* different kinds of objects, by employing a five-fingered (and six motors) robotic hand, mounted onto an orthopaedic splint designed for able-bodied users (hereafter, prosthetic hand emulator). The proposed experiments were aimed at addressing two key objectives. The first objective was to understand the effectiveness of the approach, i.e., whether it is possible to drive the hand in real-time, with reasonable performance, in different grasps. The second objective was to investigate the intuitiveness, indirectly, by using quantifiable performance metrics and therefore to assess statistical differences among these ones throughout consecutive experiments.

In the following pages the experimental setup, composed of the prosthetic hand emulator, the two degrees of freedom EMG controller and the PCA-based algorithm, is described. Finally, a description of the performed experiments is provided and the collected results are presented and discussed to be useful for future developments.

## Methods

**Figure 1:**
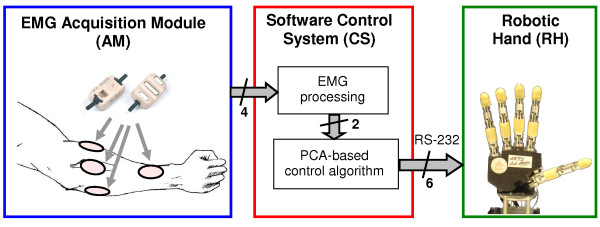
**System overview.** The experimental setup included the EMG acquisition module (AM, with four active electrodes placed on the user’s forearm and an acquisition board) and the software control system (CS), which ran on a laptop and was interfaced with the hand (RH) via serial port. The CS acquired and decoded the four EMG signals to generate two independent input signals; these were fed into the PCA-based algorithm that generated and sent the six motor control commands to the robotic hand.

The system here employed consisted of 3 modules: the robotic hand (RH), mounted onto an orthopaedic splint designed for able-bodied users, the EMG signals acquisition module (AM) and the software control system (CS) (cf. Figure 1).

### Prosthetic hand emulator

**Figure 2:**
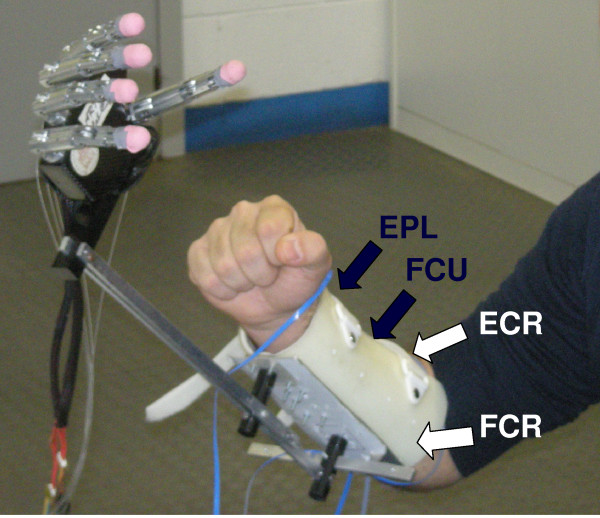
**Prosthetic hand emulator and electrodes positioning.** Experimental set-up showing the prosthetic hand emulator and the four EMG sensors on the targeted muscles: the *flexor* (FCR) and *extensor carpi radialis* (ECR), the *extensor pollicis longus* (EPL) and the *flexor carpi ulnaris* (FCU).

The robotic hand used for assessing the feasibility of the approach was a modified and simpler version of the CyberHand [27], assembled onto an orthopaedic splint which allowed any reaching movement not involving the wrist, as in Panarese *et al.*[28] (cf. Figure 2). The hand is anthropomorphic, right-handed, human-sized, with five independent underactuated fingers able to mechanically adapt on objects. Hand actuation is achieved by means of six electrical motors: five are employed for independent flexion/extension of each finger, and the sixth one for thumb abduction/adduction. The hand is able to perform the three main functional grasps defined in Iberall & Arbib’s grasp taxonomy: power, precision and lateral grasps [29]. However, as mentioned in our previous work [21], grasp stability for precision grasps is only allowed within a certain force/position workspace of the fingers due to instability problems; therefore, to perform a stable precision grasp (i) accurate positioning of the fingers and (ii) accurate force level on the object are required. A microcontroller-based hierarchical architecture implementing fingers position control is embedded in the hand and is activated by external commands from a standard RS-232 communication bus. A detailed description of the hand can be found in [27].

### EMG signals acquisition module

**Figure 3:**
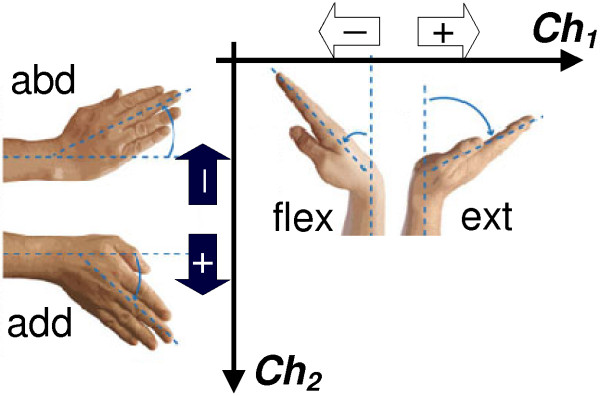
**Two DoFs control signal generation.** Wrist movements re-mapped into *Ch*_*1*_ and *Ch*_*2*_ signals variations, used to generate input commands for the PCA-based algorithm. Extending (ext) or flexing (flex) the wrist affected the input control signal *Ch*_*1*_. Adduction (add) and abduction (abd) movements influenced *Ch*_*2*_.

EMG signals were acquired by means of active surface electrodes, regularly used in myoelectric prostheses and commercially available (Myobock electrodes 13E200=50, Otto Bock Healthcare Products GmbH, Wien, Austria): signals conditioning (adjustable amplification—in this case set to 6—and low-pass filtering B = 90–450 Hz) is performed on-site by the electrode hardware itself, and for this reason in prosthetics they are generally referred as *EMG sensors* (instead of simple electrodes). Two pairs were individually adjusted and placed on the subjects’ forearm in order to pick-up independent myoelectric signal pairs generated by the activity of the following antagonist muscles: the *flexor* (FCR) and *extensor carpi radialis* (ECR), the *extensor pollicis longus* (EPL) and the *flexor carpi ulnaris* (FCU) (cf. Figure 2). It is known that wrist flexion and extension are demanded to FCR and ECR, while the other two muscles are involved respectively in wrist abduction (EPL) and adduction (FCU) movements [30]. The mutual interference between the activity of the four muscles did not represent a significant problem, due to accurate individual positioning of the EMG sensors [31]. The underlying objective was indeed to develop a myoelectric joystick (Figure 3), i.e. a two differential channels controller using EMG signals from muscles that move the wrist (similarly to [31-33]). EMG sensors were connected to an acquisition board (NI-DAQ USB-6211, National Instruments Corp., Austin, TX, USA) which communicated with the laptop that run the decoding algorithms.

### Software control system

**Figure 4:**
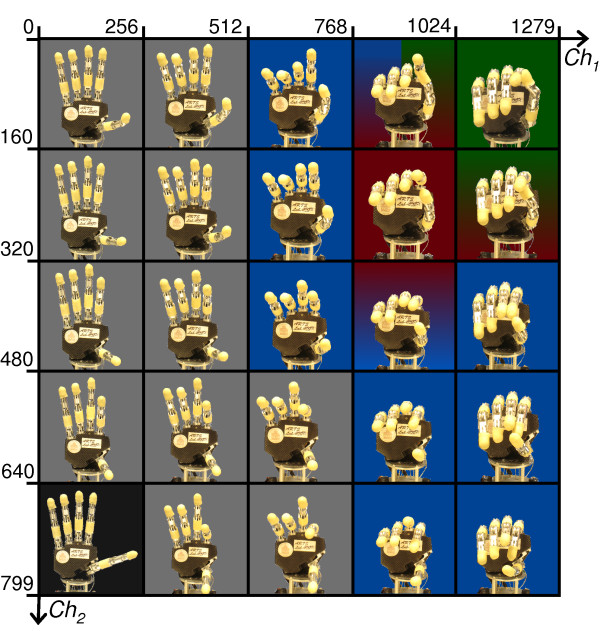
**CyberHand postures distribution.** CyberHand postures over the *Ch*_*1*_*,Ch*_*2*_ input signals plane, sampled using a 5×5 grid. Blue, red or dark green backgrounds are used to denote areas corresponding to those hand configurations which are functional for achieving respectively a power, precision or lateral grasp. Faded colours are used to indicate areas where more than one grasp type could be achieved. A black background denotes the open-hand neutral position.

A C-written application was developed using LabWindows-CVI (National Instruments) and run on the laptop connected to both the EMG acquisition module and to the hand. The control system decoded and converted the subjects’ 2-DoF wrist contractions (flexion/extension and adduction/abduction) into hand posture control commands, implementing the algorithm based on PCA previously presented by the authors in [21]. At a glance this algorithm reverted the PCA concept and allowed to drive the dexterous 6-motor hand in a pre-defined hand-posture workspace with just two independent control inputs (in this case the 2-DoF wrist movements); the pre-defined hand-posture workspace is illustrated in Figure 4[21]. The picture shows a discrete grid representing the two inputs and a sample of the postures assumed by the hand (corresponding to such inputs), i.e., the two-to-six mapping. The map denotes that some areas (i.e. some input combinations) are more functional for certain grasp types rather than others. The pre-defined workspace of the hand was actually the result of the analysis of postural data directly collected from the six position sensors in the hand, while performing a multitude of grasps (cf. Appendix A and [21]).

In this study the two control inputs were obtained from effective myoelectric signals picked-up in real-time on the forearm of able-bodied subjects wearing the prosthetic hand emulator, so that they could control (moving their wrists as shown in Figure 3) the posture of the hand in the workspace (in Figure 4), and hence grasp objects. As shown in our previous work, the first input *Ch*_*1*_ (wrist flexion/extension) mostly influenced fingers flexion/extension (horizontal axis in Figure 4) whereas the second input *Ch*_*2*_ (wrist adduction/abduction) influenced thumb rotation (vertical axis in Figure 4). For the sake of clarity, the mathematical description of the myoelectric joystick, of the hand-posture workspace and of the PCA-based controller are described in Appendix A.

### Experimental setup and protocol

Two groups of volunteers composed of six able-bodied subjects each (3 males and 3 females each, whose average age was 25.5 ± 1.8 years old), all with dominant right hand (same as the artificial hand) and naïve to EMG control, were enrolled in this study. Subjects in both groups (G1 and G2) were asked to execute the simple task of grasp, lift, transport and release an object operating in real-time the CyberHand through the myoelectric PCA-based controller introduced above. Twenty different objects were chosen (cf. Table 1), proportionally representing the percentages of power, precision and lateral grasps used in activities of daily living (ADLs) [34].

**Table 1:** Objects used in the experimental trials

**Object**	**Shape**	**Size [mm]**	**Grasp Type**
Paper roll	cylindrical	*d* = 47; *h* = 225	power
Spray	cylindrical	*d* = 52; *h* = 140	power
2 l bottle	cylindrical	*d* = 90	power
0.5 l bottle	cylindrical	*d* = 65	power
Coke tin	cylindrical	d = 65; h = 110	power
Twine roll	cylindrical	*d* = 60; *h* = 80	power
Cigarette pack	parallelepiped	20 × 55 × 85	power
Torch	cylindrical	*d* = 35; *h* = 165	power
Golf ball	spherical	*d* = 40	precision
Plastic sphere	spherical	*d* = 33	precision
Soft rubber ball	spherical	*d* = 50	precision
Electric adapter plug	cylindrical	*d* = 43; *h* = 40	precision
Plastic cube	cube	*L* = 30	precision
Paperclips pack	parallelepiped	70 × 38 × 15	precision
CD	circular	*d* = 120	lateral
Single CD case	rectangular	140 × 125 × 5	lateral
Pad-per-hole perfboard	rectangular	*h* = 2	lateral
Audio-cassette case	parallelepiped	108 × 67 × 16	lateral
Business card	rectangular	*h* = 1	lateral
Post-it notes package	parallelepiped	78 × 127 × 8	lateral

In the Size column, *d* stands for *diameter* and *h* for *height*.

Each subject was seated on a chair in front of the object to be grasped, which was placed (always in this same position) on a desk. The prosthetic hand emulator was fastened to the right forearm of the subject as shown in Figure 2.

At the beginning of the experiments, each subject wearing the system was asked to perform powerful flexion/extension and abduction/adduction movements of his/her right wrist (about 2 s for each contraction), while the corresponding EMG signals were recorded. This simple procedure was used to calibrate the CS with the subject’s muscular activity: the maximum EMG amplitude value for each channel was identified and used to set *T*_*i,1*_ and *T*_*i,2*_ noise thresholds (e.g., 1/4 of peak value) (cf. Eq. 5, Appendix A).

Starting with the artificial hand completely opened, after an initial audio cue, each task (i.e. moving the target object from its initial position on the desk to a final one, fixed 50 cm far on the same desk) started. Completion and grasping times were measured using a standard chronometer (activated after the starting cue and finally stopped once the hand returned in the open posture, after object release).

There were two sets of 20 objects for each subject and, in both sets, the target objects were presented in random order. The experimental task was the same for both G1 and G2, whereas the protocol differed. Subjects in G1 were given precise instructions on how to control the hand (i.e., how the 2-DoFs EMG joystick worked, how contractions were mapped into hand postures, how to flex muscles to obtain a defined grasp) and a twenty minutes training session (not recorded), in which they were free to train and control the CyberHand with their EMGs and to grasp different objects as they wished. After that, the experimental trials began. Subjects in G2, instead, were given only simple instructions (i.e., which muscles to contract) with no time for initial practicing, but were asked to perform the experiment on three consecutive days. The different experimental protocols for the two groups were aimed at assessing two complementary hypotheses on the intuitiveness and effectiveness of the system. The first hypothesis (assessed through G1 subjects) was that a short practice after detailed instructions would be sufficient to achieve good performance in grasp tasks. The second hypothesis (assessed through G2 subjects) was that the controller was intuitive enough that, even with no precise instructions, performance would improve with practice anyway.

Three metrics were used to quantify the quality and performance of grasp: (1) the task-completion rate (CR), defined as the percentage of correctly executed tasks (i.e. reach, grasp, transport and release the object without slippage); (2) the object-grasp time (*Tg*), defined as the time taken to correctly grasp (in subjects’ opinion) the object, from audio cue to the achievement of a firm grasp and confident object lift; (3) the task-completion time (*Tc*), defined as the time spent to perform the whole task (object grasp, transport and release, starting from the audio cue). These metrics were based on similar studies [14,35].

Statistical differences among experimental task-completion rates and times were evaluated using the Friedman test [36]. Non parametric statistics was used as the data set did not pass tests for normality. A level of p < 0.05 was selected as the threshold for statistical significance. In addition, in this study we assumed that results achieved in two consecutive sets on the same day were not statistically different. Statistical analyses were performed using MatLab (The MathWorks, Natick, MA, USA) scripts.

## Results

The six subjects enrolled in G1 performed 40 grasps each (20 objects, 2 repetitions) on a single day, for a total of 240 trials. The six subjects in G2 performed 120 grasps each in three days, for a total of 720 trials.

1) The percentage of completed tasks and 2) time spent to grasp and complete the task were used to evaluate the control system usability and how easy to learn was the re-mapping of muscular contractions into hand movements by subjects naïve to EMG control. Therefore, the evolution throughout the three days of these metrics for subjects in G2 and their comparison with group G1 performance were analyzed.

Each experimental session (two sets of grasps, including set-up and calibration) lasted about 60 min. As expected, the majority of time was spent to grasp the object (i.e., voluntarily control the hand in the prehensile pattern), after which subjects were able to transport and release it within few seconds and with minimal effort. The primary cause of trial failure (83% of total fails) was the object fall during the grasping phase, especially for small objects requiring precision grasps. In some cases (17%), the object fell during its transportation from the initial to the target point on the desk, due to a poorly stable grasp.

### Task-completion rates

**Figure 5:**
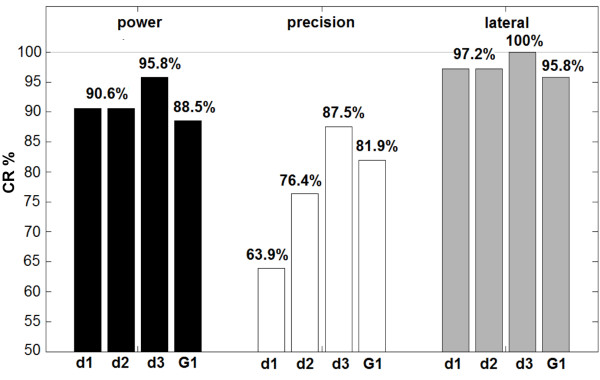
**Results: average task-completion rates.** Task-completion rates for group G2 on day 1–3 (d1–d3) and for group G1, considering both sets for each object. Black bars represent power grasps, white bars refer to precision grasps and light gray ones to lateral grasps.

The task-completion rate, for subjects in G2 on day 1, 2, 3 and for subjects in G1, is presented in Figure 5. Each bar representing power grasps (in black) includes 96 samples (8 objects, 2 repetitions, 6 subjects), precision grasps (in white) 72 samples (6 objects) and lateral grasps (light gray) 72 samples (6 objects).

The visible improvement in performance throughout the three days was not always confirmed by the Friedman test, as task-completion rates across days and subjects (G2) revealed to be statistically different only for precision grasps (p = 0.015) but not for power (p = 0.495) and lateral (p = 0.222) grasps.

### Object-grasp and task-completion times

**Figure 6:**
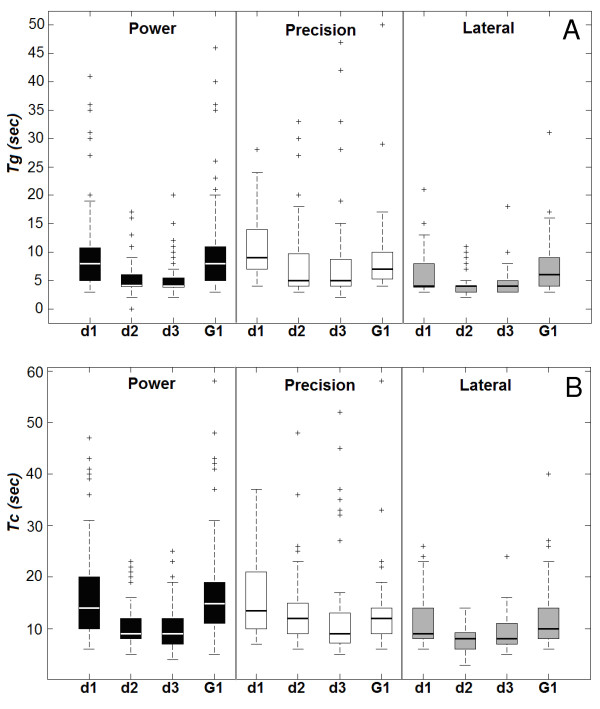
**Results: object-grasp and task-completion times distributions.** Box & whiskers plots representing (A) object-grasp time (*Tg*) and (B) task-completion time (*Tc*) distributions for subjects in G2 on day 1,2,3 (d1, d2, d3) and for group G1. Black boxes refer to power grasps, white boxes to precision grasps and the gray ones to lateral grasps. Each box is delimited by the first and third quartile values; thick horizontal lines, instead, highlight median values. Whiskers show the extent of the rest of the data, while crosses represent the outliers.

The object-grasp and task-completion time distributions for G1 and G2 on day 1, 2 and 3 are presented in Figure 6. On the average, *Tg* and *Tc* times for G2 subjects decreased along with days. The Friedman test on *Tg* revealed that there were significant differences among days for power (p < 0.001) and lateral grasps (p < 0.001) but not for precision grasps (p = 0.192). Similar results were obtained for task-completion times (power p < 0.001, lateral p < 0.001, precision p = 0.088).

**Figure 7:**
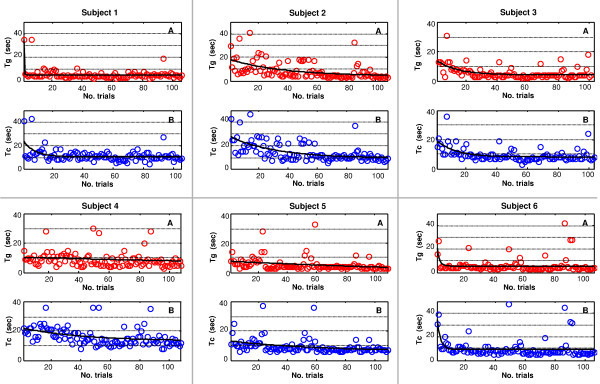
**Object-grasp and task-completion time trends throughout days for each subjects in G2.** (A) Object-grasp (*Tg*) and (B) task-completion (*Tc*) times for power, precision and lateral grasps throughout trials. Data are sorted following their temporal execution order (day 1: set 1 and 2, day 2: set 1 and 2, day 3: set 1 and 2). Each dataset is fitted with a decreasing exponential function (black curve) demonstrating the improvement in performance.

Overall, data presented in the box plots show high dispersion. Nevertheless, this large variability was caused by the differences in subjective performance and not by other variables (sets or days). This is clearly demonstrated by the graphs in Figure 7, where all task-completion and object-grasp times are plotted along with days (outlier samples mostly refer to precision grasps). Although each subject’s performance was different (i.e. subject 6 was generally faster than subject 4), all of them presented a clear improvement (*Tc* and *Tg* decrease) with time. The slope of the plotted fitting curve (i.e. a single decaying exponential function obtained by means of non-linear least squares fitting) may vary among them, but the trend is always (encouragingly) negative.

Moreover, at a glance graphs in Figure 7 show similar trends between pair wise task-completion and grasp-object times. This is confirmed by the Wilcoxon signed-rank test [37] (taking care of subtracting the mean value from each distribution) in 11 subjects out of 12, demonstrating that the transport and release phases of the task did not significantly influence the overall subject’s performance.

It should also be noted that the long times required to perform a grasp (*Tg* up to 20 s and over) should be related to the hand speed, which was very low (see the “Concluding remarks”). The minimum time required to perform stable grasps (in the three prehensile forms) was measured, resulting in 4.2 s for power grasps and 3.2 s for lateral and precision grasps. Therefore, as depicted in Figure 6 and Figure 7, day 3 results were considerably near to the best achievable ones.

## Discussion

The objective of this study was to demonstrate the feasibility of the PCA-based control algorithm, previously presented by the authors in [21], in more realistic conditions, i.e. controlling in real-time the prosthetic hand emulator with actual myoelectric signals.

Experimental trials demonstrated that the myoelectric controller worked properly and that the PCA-based approach allowed to drive—in the correct prehensile pattern—a multi-fingered, multi-DoF hand with just two differential EMG channels, i.e., with a clinically viable system. Since the approach aims to improve the usability of the prosthesis, the physical features of the grasp (e.g. *form closure*, *force closure*, *grasp stability*, etc.) were not considered as a measurement of performance, but rather we preferred to investigate usability-related metrics. Indeed, these can portray more interesting insights on the ability of the final users to learn how to control the artificial hand through the present system. Several considerations can thus be made on the performance achieved by each group.

### G1 results: initial training ensures good performance

#### CR

For subjects belonging to G1 who operated the hand in a single experimental session, after the 20 min practice, task-completion rates for power and lateral grasps were similar, also statistically (Friedman test: power p = 0.58, lateral p = 0.37), to those obtained by G2 on the three days and, importantly, considerably high (cf. Figure 5). The CR in precision grasps was also high. These results underline the importance played by the 20 min training and instructions given, effectively sufficient for gaining significant confidence with the system and for achieving excellent performance (CR = 89% on the average).

#### Tg and Tc

With regard to time metrics, results for group G1 are closer to those obtained by G2 on the first day, rather than those on the second or third day (cf. Figure 6). As described below, this is probably due to the fact that improvements in time metrics are visible only after several trials.

### G2 results: performance improves with time

Observing results achieved by group G2, which underwent a three days experimental session, it is possible to highlight issues on the subjects’ performance evolution.

#### CR

Generally, the CR increased along with days (cf. Figure 5). However, improvements were statistically significant only for precision grasps (p = 0.015), as these are (in general and especially in the beginning, with no particular instructions on how to perform them) the most difficult ones, due both to the mechanical features of the hand and to the shape of the grasped object (often spherical) [21]. Therefore, performance improvements were particularly pronounced (i.e. the height of precision grasp bars in Figure 5 significantly increase), underlying that subjects learned how to finely operate the hand thanks to practice.

For power and lateral grasps, which are instead simpler, a high performance was already achieved on day 1, and thus CR displayed just a slight increase (not statistically significant).

#### Tg and Tc

Concerning time analyses, results revealed a very high dispersion; this was mainly due to inter-subject variability and would decrease if considering the six subjects separately (Figure 7). Subjective time trends, together with average task completion rates, demonstrate that those subjects who underwent a three days experimental session soon learned how to functionally control the artificial hand, improving their performance.

*Tg* (and *Tc*) values significantly decreased throughout days for power and lateral grasps but not for precision grasps, which anyhow (importantly) did not increase. In this last case, their persistent high variability was probably affected by the intrinsic complexity and effort required to control the hand in such posture.

#### Precision grasps accuracy

The time-improving grasp accuracy for precision grasps was clearly visible for the operator who supervised the experiments: on the first day, almost all subjects exploited the inherent dexterity of the PCA-controller, trying to perform grasps with the minimum effort. They mostly used only one DoF (i.e., the one mainly controlling fingers flexion/extension) which was good enough to grasp all objects, even if approximately. On day 2 and 3 instead, they controlled the hand more accurately.

**Figure 8:**
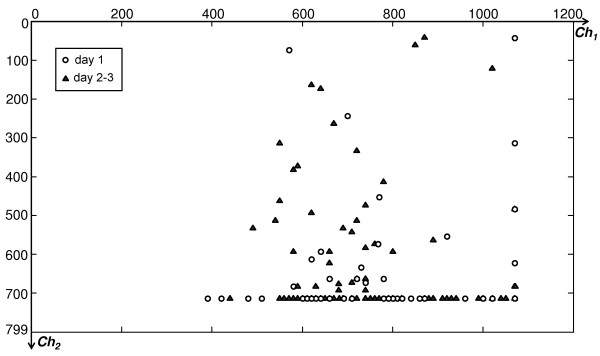
**Control signal values corresponding to the reaching of stable precision grasps.** Precision grasp points distribution over the *Ch*_*1*_*,Ch*_*2*_ plane for all subjects in G2. Circles denote grasps on the first day, triangles correspond to grasps on day 2 and day 3.

This can be demonstrated by Figure 8, which represents in the *Ch*_*1*_*,Ch*_*2*_ plane (the two signals that modulate the two principal components) the values reached when stable precision grasps were achieved. Circles refer to day 1 (naïve subjects), whereas triangles refer to day 2 and 3 (when subjects were supposed to be trained). It is immediate to observe that the majority of day 1 samples are distributed along the bottom horizontal axis (where *Ch*_*2*_ = *max*(*Ch*_*2*_)), that corresponds to a rough powerful adduction of the wrist. On day 2 and 3 the control became finer: this is denoted by the increased concentration of points spread over the *Ch*_*1*_,*Ch*_*2*_ plane, meaning that subjects paid more attention in operating the system (not just making *Ch*_*2*_ saturate, as on day 1). Nevertheless, importantly, the improvement in accuracy and completion rate was not paid in terms of increased object-grasp time (Figure 6a).

### Concluding remarks

Some final remarks on the setup should be provided. The relatively low speed of the hand is a parameter that certainly affected experimental outcomes. For this reason, in fact, the measured time values are definitely long (cf. Figure 6, where *Tg* are up to 20 s); however, what is interesting and worth publishing are the differences in times and trends throughout days and not absolute values. It should be noted that the minimum time required to perform stable grasps (in the three prehensile forms) was measured and resulted in 4.2 s for power grasps and 3.2 s for lateral and precision grasps. Therefore, as depicted in Figure 6 and Figure 7, day 3 results were considerably near to the best achievable ones. It is still not clear whether a faster hand would improve or worsen performance, as a more reactive system may be less controllable, especially during accurate movements. Further researches dealing with these aspects and employing a faster hardware are therefore foreseen.

The present ones are the first experiments and the viability of such system, which is mostly indicated for transradial amputees, would be in the end demonstrated by amputated subjects performing real activities of daily living. Nevertheless, it is reasonable to suppose that EMG performance in controlling a hand prosthesis—especially using four electrodes—would not significantly change with amputees using a traditional myoelectric hand (as shown in [13] and [14]). In particular, if the amputation level ranges from wrist disarticulation to transradial in the lower third, we can assume that results would not be significantly different, as the muscles targeted in this study would be preserved. If the amputation instead is more proximal, e.g. short below the elbow, four independent EMG sites should be found and their positioning should be assessed in each different case. Even with a transhumeral amputation the present system could still be implemented, but in this case the setup should be changed (e.g. placing electrodes sites on the shoulder and breast muscles) and results would probably be different.

## Conclusions

In this work, we demonstrate for the first time that a PCA-based controller can be successfully combined with a two DoFs (4 electrodes) EMG acquisition system and easily used by able-bodied participants to control in real-time the prehension of a five-fingered six-motorized artificial hand. The experimental results of the trials described in this paper demonstrate that this bio-inspired myoelectric interface and control system has the great potential to become a usable means for amputees by achieving both ease of use and dexterous functionality, and by allowing them at last to manage their hand prosthesis in a more intuitive and natural way.

## Appendix A

### Myoelectric joystick

The myoelectric joystick was implemented as follows. Signals generated by the antagonist *extensor* and *flexor carpi radialis* (*s*_*1,1*_ and *s*_*1,2*_, wrist extension/flexion) were combined to obtain the first DoF (*Ch*_*1*_ signal) of the control input; signals from the *flexor carpi ulnaris* and the *extensor pollicis longus* (*s*_*2,1*_ and *s*_*2,2*_, wrist adduction/abduction) were used to obtain the second DoF (*Ch*_*2*_ signal) (cf. Figure 3). Signals were sampled at 1 kHz, and integrated over 20 ms non-overlapping windows:

(1) $$S_{i,j}\left(k\right)=\sum_{t=Nk}^{Nk+N-1}s_{i,j}\left(t\right)\;i=1,2\text{,}\;j=1,2$$

where *t* represents sampled time (*t* = 0,…,*t*_*n*_, being *t*_*n*_ the time of acquisition end), *N* is the number of samples included in 20 ms (i.e. 20, if sampling at 1 kHz) and *k* is an integer index:

(2) $$k=0,1,2\text{,}\ldots \text{,}\;floor\left(\frac{t_{n}+1}{N}\right)-1$$

(*floor* stands for “round to the nearest lower integer value”). Index *i* is used to denote the muscles pair (i.e., FCR and ECR or EPL and FCU), while *j* refers to the muscles inside the pair.

Two combined signals *Sx*_*i*_*(k)* (one for each muscles pair) were then computed by multiplying the maximum between the integrated signals of each two antagonist muscles (*S*_*i,j*_*(k)*) by an empirical corrective factor *F*_*i,k*_:

(3) $$Sx_{i}\left(k\right)=F_{i,k}\cdot \max_{j}\left(S_{i,j}\left(k\right)\right)\cdot sign\left(S_{i,1}\left(k\right)-S_{i,2}\left(k\right)\right)\text{,}$$

with *k* varying as in (2).

Finally, signals *Ch*_*1*_ and *Ch*_*2*_ (i.e. the outputs of the EMG processing module) were computed by simple integration (accumulation):

(4) $$Ch_{i}\left(k\right)=Sx_{i}\left(k-1\right)+Sx_{i}\left(k\right)\text{.}$$

The empirical corrective factors *F*_*i,k*_ depended on the four EMG signals as follows:

(5) $$\begin{array}{l} F_{i,k}=0\;if\;\left(S_{i,1}(k)<T_{i,1}\right)\wedge \left(S_{i,2}(k)<T_{i,2}\right)\\ \left\{ \begin{array}{l} F_{1,k}=1\\ F_{2,k}=0.8 \end{array}if\;\left(M(k)\in \left\{S_{1,1}(k),S_{1,2}(k)\right\}\right)\wedge \left(M(k)>1.5\cdot S_{2,j}(k)\right)\right.\\ \left\{ \begin{array}{l} F_{1,k}=0.6\\ F_{2,k}=1 \end{array}\;if\;\left(M(k)\in \left\{S_{2,1}(k),S_{2,2}(k)\right\}\;\right)\wedge \left(M(k)>1.5\cdot S_{1,j}(k)\right)\right.\\ F_{i,k}=1\;otherwise \end{array}$$

where *j = 1,2*; *T*_*i,1*_ and *T*_*i,2*_ were threshold values (experimentally assessed), below which signals were considered as noise and, each time, *M(k)* was the maximum value among the four signals *S*_*i,j*_*(k)*.

In practice, every 20 ms the highest integral value among the four (e.g. *S*_*1,1*_*(k)*) was compared with the ones generated by the other pair of muscles (in this example, *S*_*2,1*_*(k)* and *S*_*2,2*_*(k)*) and if it was sufficiently greater (i.e. *S*_*1,1*_*(k) > 1.5·S*_*2,1*_*(k)* and *S*_*1,1*_*(k) > 1.5·S*_*2,2*_*(k)*), then the corrective factor for the “losing” pair of muscles was set to a number between 0 and 1 (i.e. 0.8 or 0.6 vice-versa). The corrective factor for the “winning” pair was always set to 1, in order to totally reflect the maximum muscular contraction into a variation of the corresponding *Ch*_*i*_ signal; whereas, if muscular activity was below the thresholds (i.e., considered as noise), it was set to zero.

The two signals (DoFs) *Ch*_*1*_ and *Ch*_*2*_ were finally fed into the “inverse PCA” algorithm (presented in [21] and briefly resumed below) to compute the six end-point position values for the control of the CyberHand.

As regards the empirical factors used, the need to introduce them emerged in preliminary investigations, while plotting the two channels *Ch*_*1*_ and *Ch*_*2*_ on the *x, y* axes of the monitor screen, i.e. converting them into coordinate values of a 2D cursor. During these first trials, the obtained cursor movements were not fluent; in particular subjects had difficulties in moving the cursor along oblique paths, since the contributions of the two muscles pairs to the two signals were not well balanced (each time the “winning pair” too strongly prevailed on the other pair). Thus, different *F*_*i,k*_ values were used to correct them, trying to enhance each time the action of the winning muscles pair and to lower the other one’s, but without completely cancelling it. Moreover, signals generated by *flexor* and *extensor carpi radialis* were found to be significantly higher than those produced by the other pair [38]; this is why, when the wrist adduction/abduction (usually weakly) prevailed, an even lower corrective factor (e.g. 0.6) was required to attenuate those strong muscles contribution.

Hence, the corrective factors finally chosen permitted: first, to compute a 2-DoFs, continuous (50 Hz rate), and independent (synchronous and not exclusive) output (as both channels, if above thresholds, were always computed); second, to match the cross-effects of different pairs of muscles (by setting different corrections to the losing pair, i.e. 0.6 or 0.8).

Being the corrective factors subject-dependent, they had to be properly calibrated for each user; in this study however, the values reported above demonstrated to be a good trade-off for all subjects involved. If electrodes were placed on other muscles (which might behave differently and have different strengths) these values should be properly re-tuned. Noise thresholds *T*_*i,j*_ (one for each recorded signal) also required subjective tuning and were calculated in this work during the calibration procedure preceding each experimental session, as a fraction (1/4, found empirically) of the maximum value of the recorded EMG signal.

### PCA-based controller and hand-posture workspace

Being inspired by the work of Santello *et al.*[20], in our previous paper [21] we presented a PCA-based controller able to control some level of dexterity in multi-DoF hands by means of just a 2-DoF control input.

The PCA algorithm [39] is briefly described hereafter. Having a dataset matrix of hand postures (where each datum consists of N position values), the eigenvectors of its covariance matrix (NxN) are the PCs and the related eigenvalues are the PCs weights, which represent the amount of data variance the PCs can explain. By multiplying the dataset matrix by the PCs matrix (whose columns are the PCs vectors ordered in descending order according to their weights) a new dataset is obtained, where variables are uncorrelated. PCA can also be used for dimensionality reduction, since, if the last PCs have a low weight, they can be neglected, and each datum in the new set will be represented by a reduced number of variables.

In the specific case of the CyberHand, the analysis of postural data directly collected from the hand six position sensors (meaning that the original data dimension is N=6), while performing a multitude of grasps with different objects, demonstrated that the first two PCs can account for almost 90% of these data variance. Thus, inverting the PCA algorithm and neglecting the low-weight PCs, these 2 PCs can be used to transform a 2D input (*Ch*_*1*_, *Ch*_*2*_) into its 6D original counterpart (*Out*_*1*_,…*Out*_*6*_), which consists of six position values for the hand fingers:

(6) $$\left[\begin{array}{cccc} \vec{PC_{1}} & \vec{PC_{2}} & \ldots & \vec{PC_{6}} \end{array}\right]\cdot \left[\begin{array}{c} Ch_{1}\\ Ch_{2}\\ 0\\ \ldots \\ 0 \end{array}\right]=\left[\begin{array}{c} PC_{1,1}\cdot Ch_{1}+PC_{2,1}\cdot Ch_{2}\\ PC_{1,2}\cdot Ch_{1}+PC_{2,2}\cdot Ch_{2}\\ PC_{1,3}\cdot Ch_{1}+PC_{2,3}\cdot Ch_{2}\\ \ldots \\ PC_{1,6}\cdot Ch_{1}+PC_{2,6}\cdot Ch_{2} \end{array}\right]=\left[\begin{array}{c} Out_{1}\\ Out_{2}\\ Out_{3}\\ \ldots \\ Out_{6} \end{array}\right]$$

where *PC*_*i,j*_ is the *j*-th dimension of the *i*-th principal component (column) vector *PC*_*i*_.

In practice, the calculated PCs matrix, allows to transform two independent inputs *Ch*_*1*_ and *Ch*_*2*_ (e.g. the mouse coordinates on a PC screen) to a defined workspace of hand postures (cf. Figure 4).

## Competing interests

CC hold shares in Prensilia S.R.L., the company that manufactures robotic hands as the one used in this work, under the license to Scuola Superiore Sant’Anna.

## Authors’ contributions

GCM and CC have full access to the data in the study and take responsibility for the integrity of the data. Study concept and design, software development, acquisition and interpretation of data, drafting of the manuscript: GCM and CC. Design of the low-level control of the CyberHand: CC and MCC. Critical revision of the manuscript for important intellectual content: MCC and GM. All authors read and approved the final manuscript.
